# Native Aortic and Mitral Valve Endocarditis Caused by Nontoxigenic Corynebacterium diphtheriae in a Young Male Patient: A Case Report

**DOI:** 10.7759/cureus.113921

**Published:** 2026-08-04

**Authors:** Thomas Oswald, Cara Hanley, Jim Frem, Gillian Jones, Michael Lewis, Rachael James

**Affiliations:** 1 Intensive Care Medicine, University Hospitals Sussex NHS Foundation Trust, Brighton, GBR; 2 Cardiology, University Hospitals Sussex NHS Foundation Trust, Brighton, GBR; 3 Infectious Diseases, Cambridge University Hospitals NHS Foundation Trust, Cambridge, GBR; 4 Infectious Diseases, University Hospitals Sussex NHS Foundation Trust, Brighton, GBR; 5 Cardiac Surgery, University Hospitals Sussex NHS Foundation Trust, Brighton, GBR

**Keywords:** cutaneous diphtheria, epidemiology and public health, homelessness, infective endocarditis, multidisciplinary teams, recreational drug use

## Abstract

Non-toxigenic *Corynebacterium diphtheriae* strains are emerging as a cause of invasive disease, including highly virulent endocarditis, and have been associated with aggressive valvular destruction and embolic complications in reported cases without classical risk factors.

A 26-year-old man with a history of former intravenous drug use and recent inhalational drug use presented with symptoms of systemic infection. He did not have any known pre-existing cardiac disease. Blood cultures grew *C. diphtheriae*. A transthoracic echocardiogram revealed large (>1 cm) aortic and mitral vegetations associated with severe valvular regurgitation, requiring urgent surgery within three days of admission. The patient underwent prosthetic aortic and mitral valve replacement, aortic root abscess de-roofing, and tricuspid valve debridement. The patient completed four weeks of antibiotics as advised by the United Kingdom Health Security Agency (UKHSA), made a good recovery, and was ultimately discharged from the hospital.

Increasing global reports, particularly among individuals experiencing homelessness or using inhaled drugs, highlight the progressive and destructive nature of *C. diphtheriae* endocarditis, particularly in those without classical risk factors according to current diagnostic criteria. With growing clusters of infections in marginalized populations, clinicians must maintain a high index of suspicion to facilitate timely intervention and mitigate public health risks. This case reinforces the role of early recognition, coordinated multidisciplinary team (MDT) management, and collaboration with public health teams to improve outcomes.

## Introduction

Infective endocarditis (IE) is a life-threatening, multisystem disorder that commonly presents without a classical history or typical examination findings. From 2010 to 2021, there was a 40% increase in the prevalence of IE globally, with an age-standardized prevalence rate of 5.3 per 100,000 in 2021 [1]. This trend is likely associated with improved detection due to heightened awareness and enhanced diagnostic techniques, increased use of intracardiac devices and valve replacements, and an aging population. There was a 23% increase in the absolute number of deaths globally during the same time period [1]. As IE is expected to pose greater challenges in the future, it is critical that policies, control measures, and available healthcare services are optimized.

Bacteremia with *Corynebacterium* species is often attributed to skin contamination of blood culture samples and rarely represents true bacteremia, with a recent study reporting that around 8.8% of *Corynebacterium* isolates from blood cultures represented true infection [2]. Non-toxigenic *Corynebacterium diphtheriae* is an atypical cause of IE and is more commonly recognized for causing respiratory and cutaneous infections [3]. Recent clusters of IE caused by *C. diphtheriae* have been reported in the UK, South Africa, Australia, and New Zealand over the last three decades. There was no consensus regarding individual cardiac risk factors among any of these reported patient groups [4-6].

## Case presentation

**Figure 1:**
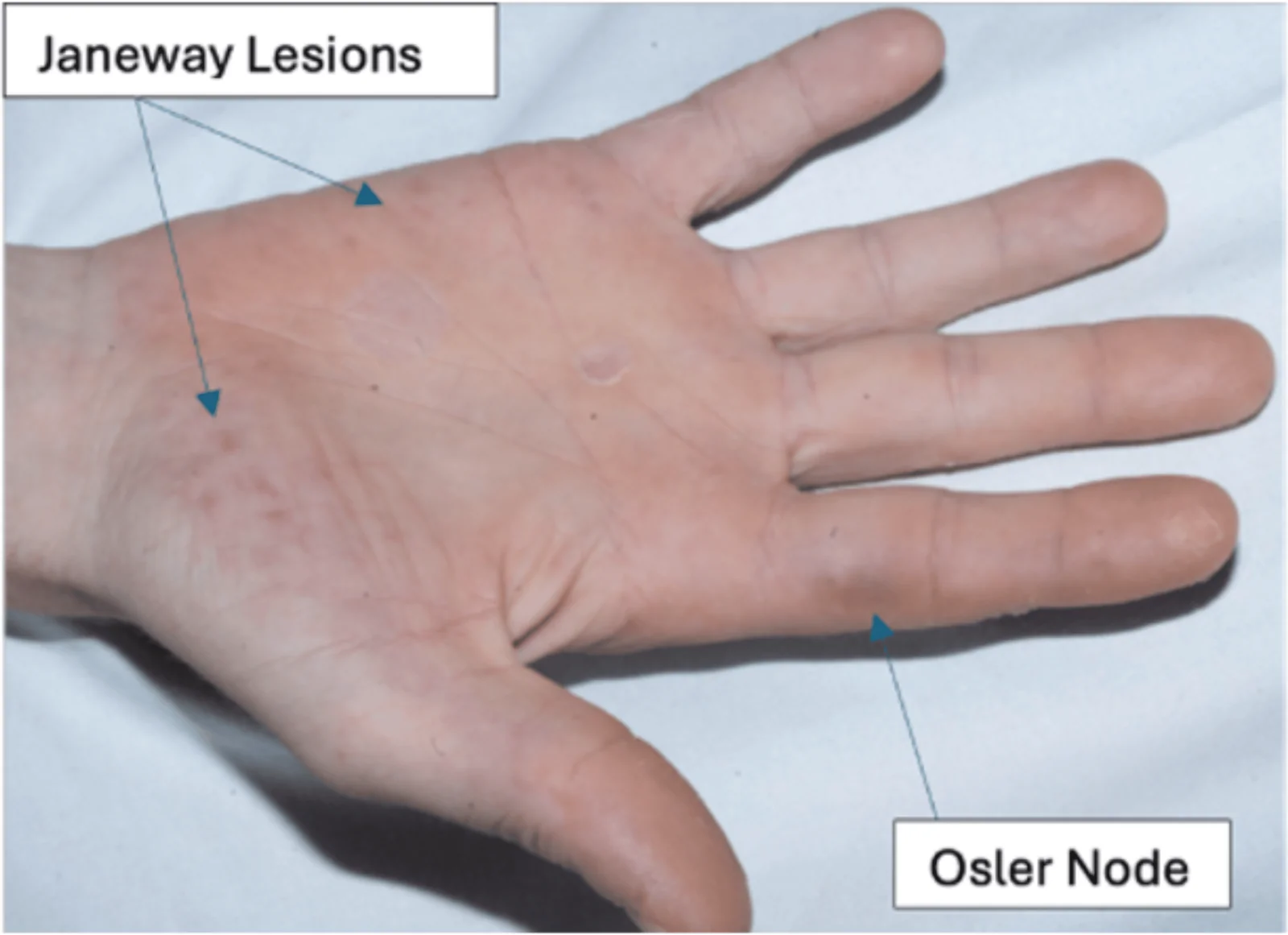
The patient's right hand showing characteristic Janeway lesions on the thenar and hypothenar eminences and an Osler node on the index finger

A 26-year-old man presented with a two-week history of generalized symptoms, including fever, diaphoresis, anorexia, and weight loss. He had a history of treated hepatitis C, asthma, and borderline personality disorder. He reported recreational drug use, with inhaled cocaine use in the last month and intravenous heroin use six months prior to admission, and was living in temporary accommodation. His initial examination revealed a collapsing pulse with an early diastolic murmur over the left sternal edge and a pansystolic murmur over the apex, with audible crackles at the right lung base. He had splinter hemorrhages on his right thumb and left middle and index fingers, as well as Osler nodes and Janeway lesions (Figure 1). There was no associated lymphadenopathy, but he had nontender splenomegaly. A Roth spot was present in the left eye on subsequent fundoscopy.

**Figure 2:**
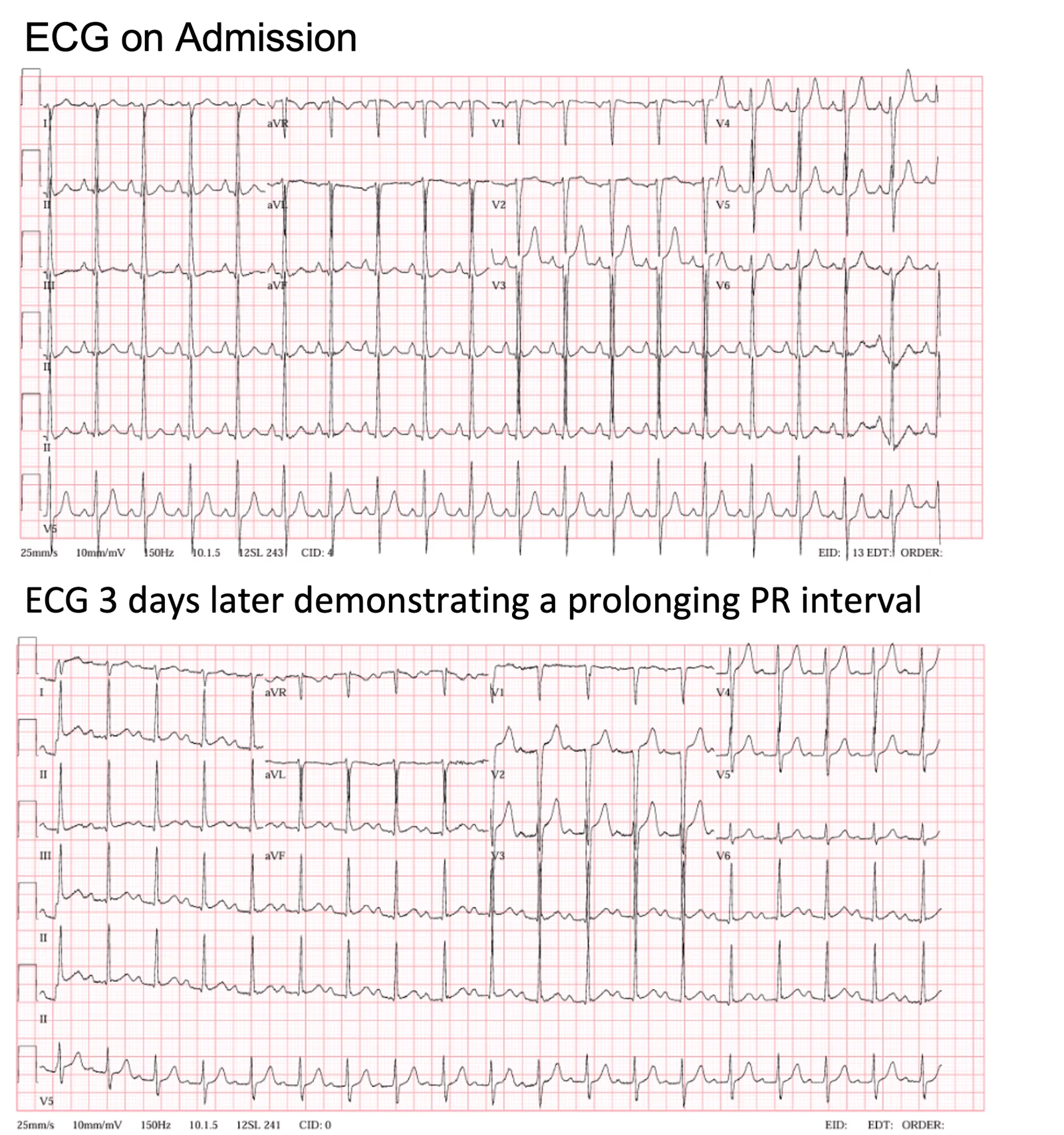
Initial ECG on admission and subsequent ECG obtained three days later, before cardiac surgery, demonstrating PR interval prolongation ECG, electrocardiogram

On admission, his heart rate was 115 beats per minute, his blood pressure was 114/49 mmHg, his temperature was 38°C, and his oxygen saturation was 99% on room air. His electrocardiogram (ECG) showed sinus tachycardia but subsequently demonstrated a prolonged PR interval (Figure 2). Blood tests revealed elevated inflammatory markers, acute kidney injury, and profound thrombocytopenia (Table 1).

**Table 1:** Initial blood test results, blood film, and urine dipstick findings on hospital admission

	Admission results with normal ranges
Hemoglobin (g/L)	128 (135-180)
C-reactive protein (mg/L)	131 (0-5)
White blood cell count (10^9^/L)	18.0 (4-10)
Neutrophil count (10^9^/L)	15.6 (86.6%) (2-7)
Lymphocytes (10^9^/L)	1.0 (5.3%) (1-3)
Platelet count (10^9^/L)	29 (150-410)
Urea (mmol/L)	16.2 (2.8-8.1)
Creatinine (μmol/L)	107 (59-104)
Sodium (mmol/L)	117 (136-145)
Potassium (mmol/L)	4.3 (3.5-5.1)
Lactate (mmol/L)	2.5 (0.5-1.6)
Blood film	Thrombocytopenia appears genuine. No clots or clumps. Neutrophilia with toxic granulation and cytoplasmic vacuoles
Urine dipstick	+3 protein; +1 blood; no other findings

**Figure 3:**
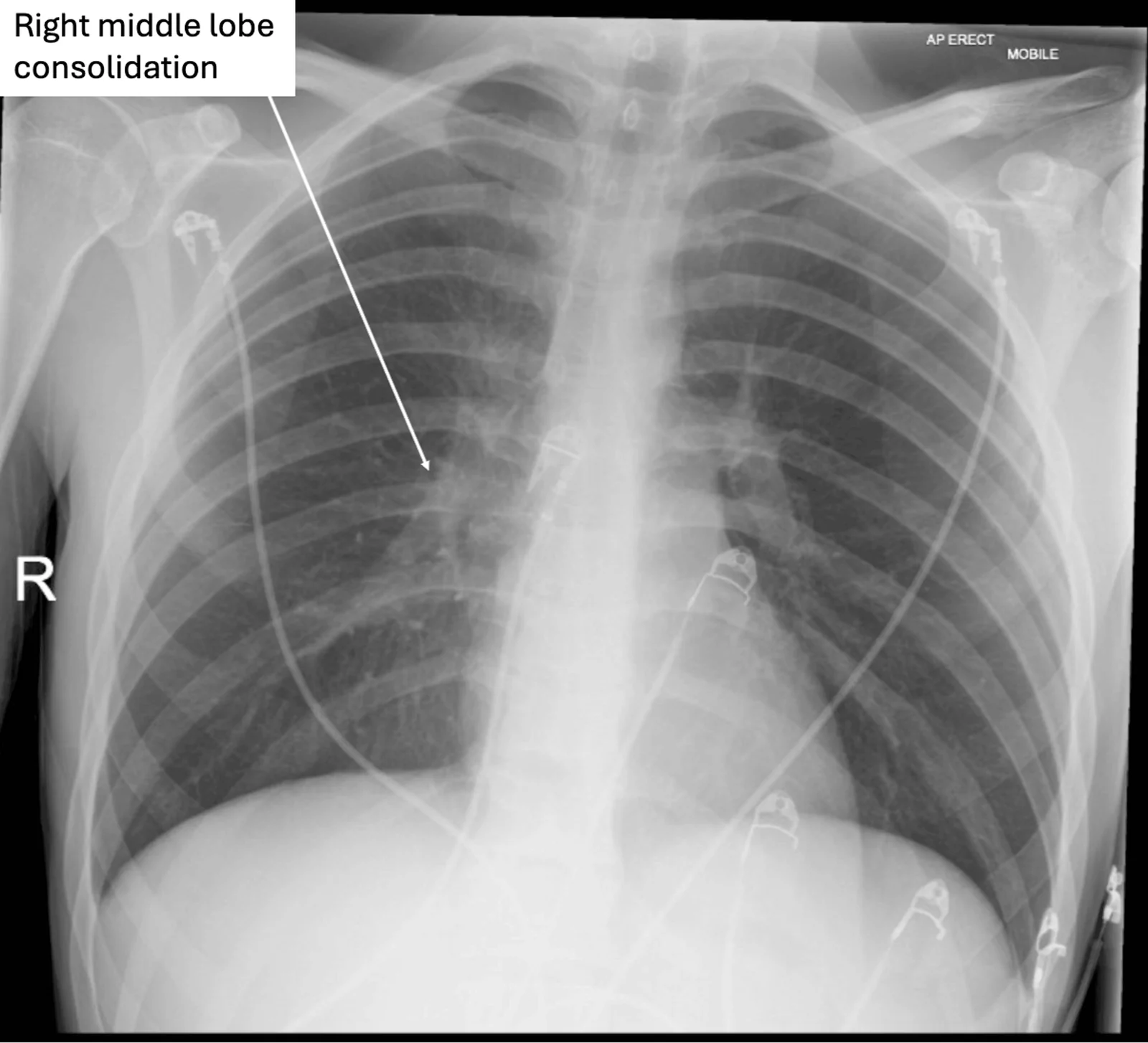
Anteroposterior chest X-ray demonstrating right middle lobe consolidation

**Figure 4:**
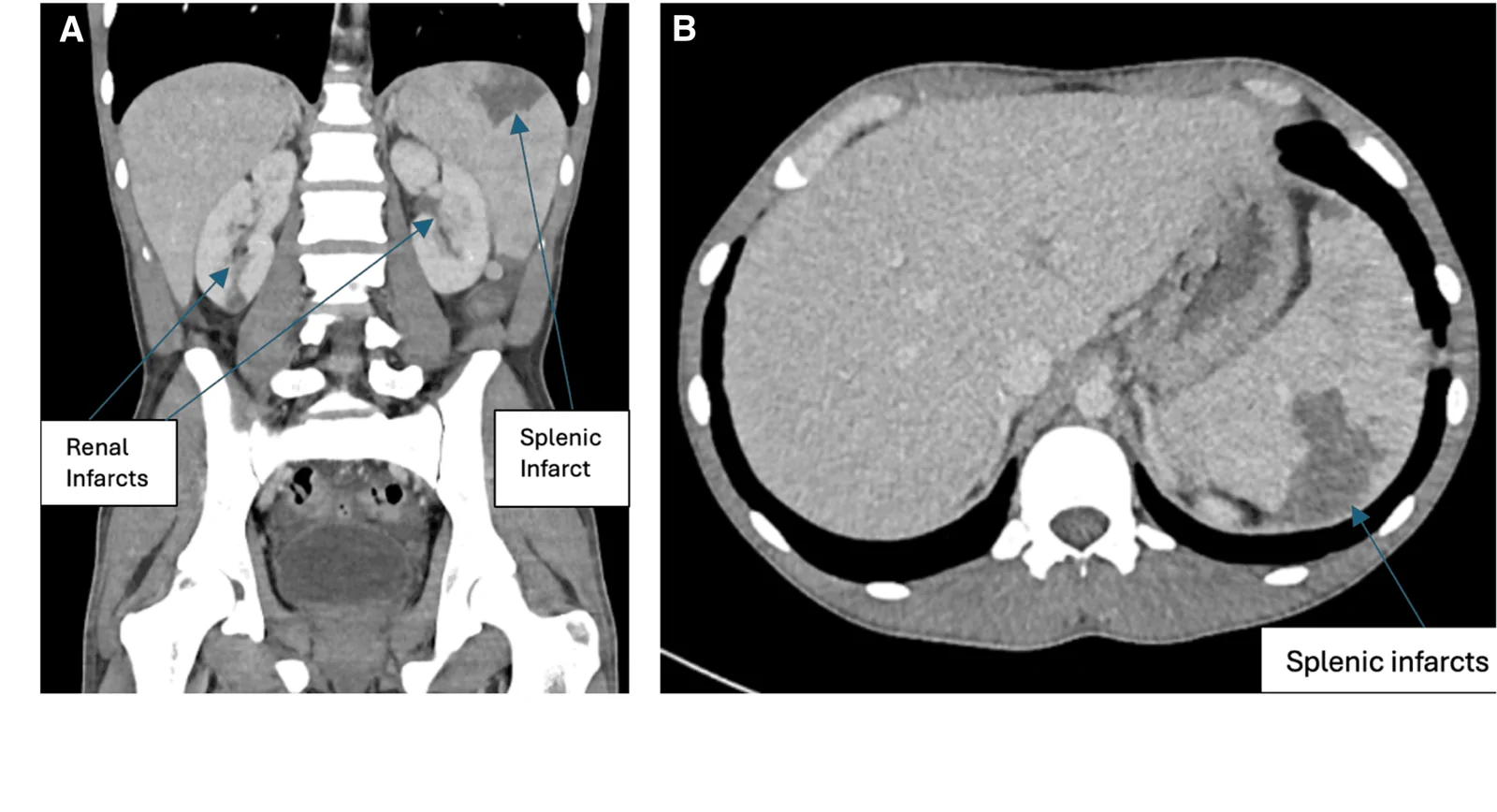
Contrast-enhanced CT of the abdomen and pelvis (A) Coronal view showing bilateral renal infarcts and multifocal splenic infarcts. (B) Axial view showing multifocal splenic infarcts.

His chest X-ray showed right-sided consolidation (Figure 3), and contrast-enhanced CT of the abdomen and pelvis showed splenomegaly with multifocal renal and splenic infarcts (Figure 4). Our local guidelines for the treatment of IE include amoxicillin, flucloxacillin, and gentamicin. In this case, the patient had a previous methicillin-resistant *Staphylococcus aureus* (MRSA) skin infection three months earlier; therefore, vancomycin and rifampin were initially added for optimal MRSA coverage in people who inject drugs (PWID). His acute kidney injury was considered prerenal, and this, together with his hyponatremia, resolved with appropriate fluid resuscitation, supporting a hypovolemic state. His thrombocytopenia improved with treatment of the underlying infection, with his platelet count rising to 136x10^9^/L within 72 hours.

**Figure 5:**
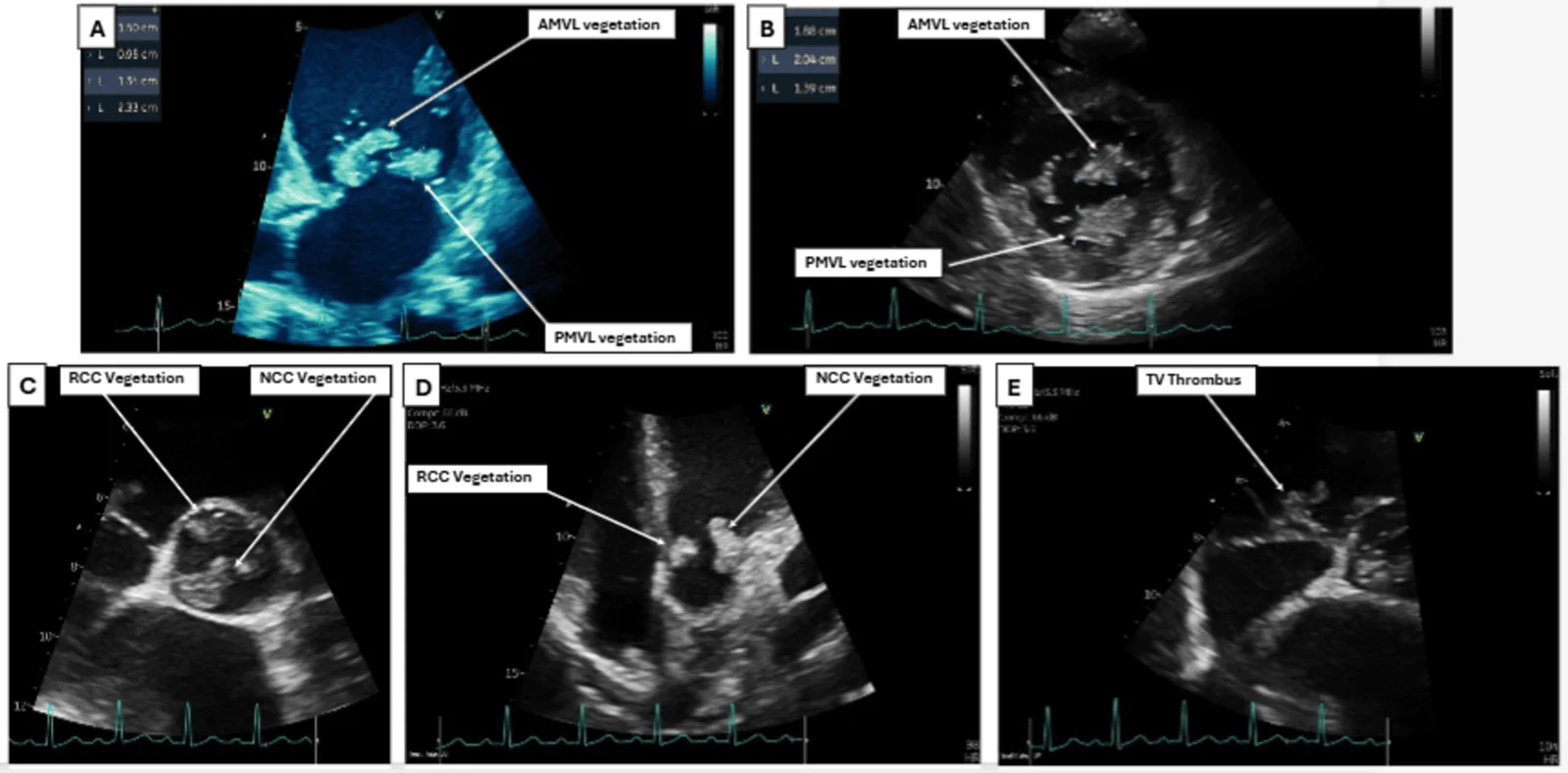
TTE images demonstrating valvular endocarditis (A) TTE A4CH view focused on the mitral valve, showing vegetations on the AMVL and PMVL. (B) TTE PSAX view at the mitral valve level, showing vegetations on the AMVL and PMVL. (C) TTE PSAX view at the aortic valve level, showing vegetations on the NCC and RCC. (D) TTE A5CH view focused on the aortic valve, showing vegetations on the NCC and RCC. (E) TTE PSAX view focused on the tricuspid valve, showing echobright material without independent mobility, consistent with thrombus. A4CH, apical four-chamber; A5CH, apical five-chamber; AMVL, anterior mitral valve leaflet; NCC, noncoronary cusp; PMVL, posterior mitral valve leaflet; PSAX, parasternal short-axis; RCC, right coronary cusp; TTE, transthoracic echocardiography; TV, tricuspid valve

**Figure 6:**
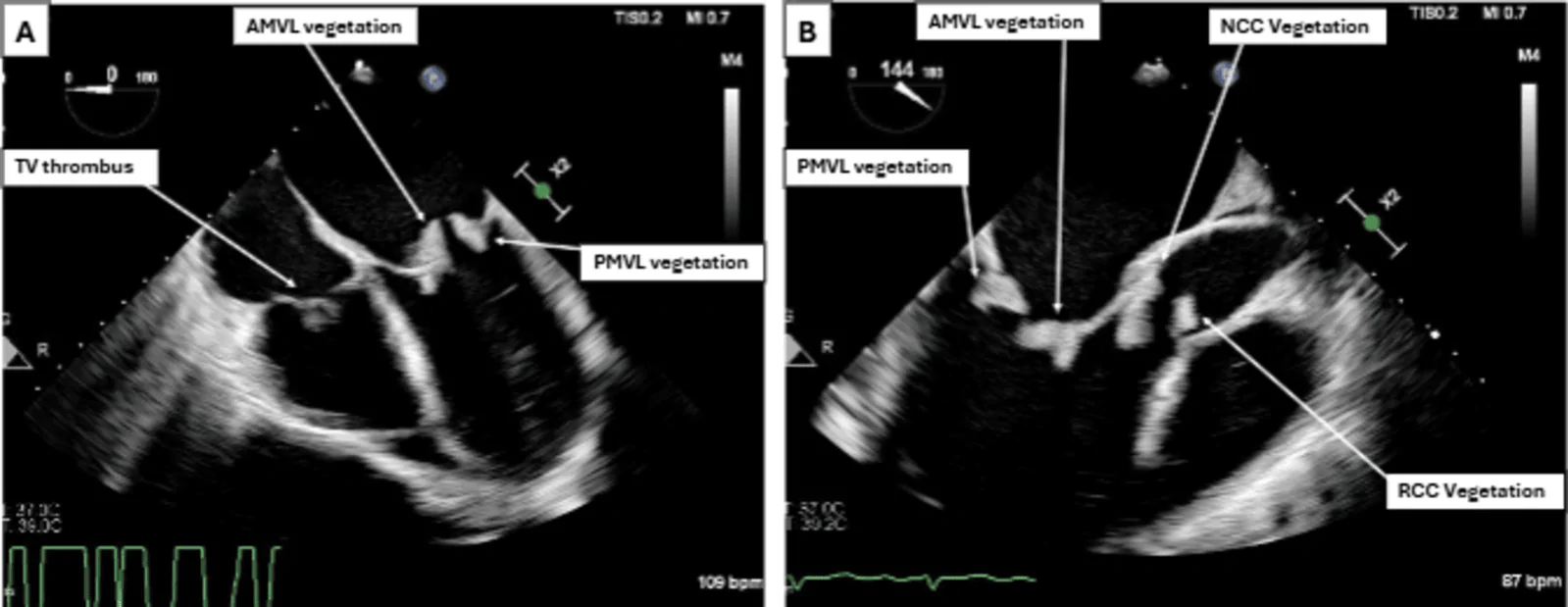
TEE images depicting valvular endocarditis (A) Mid-esophageal A4CH TEE view showing vegetations on the AMVL and PMVL, as well as masses attached to the TV with different echogenicity from the left-sided valvular vegetations, more consistent with thrombotic material. (B) Mid-esophageal long-axis TEE view showing vegetations on both the AMVL and PMVL and on the RCC and NCC of the aortic valve. A4CH, apical four-chamber; AMVL, anterior mitral valve leaflet; NCC, noncoronary cusp; PMVL, posterior mitral valve leaflet; RCC, right coronary cusp; TEE, transesophageal echocardiography; TV, tricuspid valve

As described in Figure 5, transthoracic echocardiography (TTE) showed large aortic valve vegetations attached to the noncoronary cusp (NCC) and right coronary cusp (RCC), measuring 2.33×0.83 cm and 1.06×1.15 cm, respectively, with associated severe aortic regurgitation (pressure half-time 147 ms with flow reversal in the descending and abdominal aorta). TTE also showed large vegetations on both the anterior and posterior mitral valve leaflets, measuring 2.33×1.34 cm and 1.50×0.95 cm, respectively, with associated severe mitral regurgitation (vena contracta 0.81 cm, with flow reversal into the pulmonary veins). The tricuspid valve leaflets on TTE appeared to show echobright material consistent with a possible vegetation; however, the lack of independent mobility and only trivial regurgitation were more consistent with thrombus (Figure 5). Biventricular function was preserved, and there were no other structural abnormalities. An urgent transesophageal echocardiogram (TEE) confirmed these findings (Figure 6).

Non-toxigenic *C. diphtheriae* was confirmed in one aerobic bottle in the first blood culture set and in both aerobic and anaerobic bottles in the second blood culture set, out of a total of three sets. The case was then discussed with the United Kingdom Health Security Agency (UKHSA), which advised standard barrier precautions, discontinuation of vancomycin, and initiation of benzylpenicillin for four weeks, clindamycin for four weeks, and a total of two weeks of gentamicin, counted from the date of surgery. Rifampin was discontinued after 48 hours because the patient had no foreign material, and gentamicin dosing was adjusted according to renal function with therapeutic drug monitoring. The penicillin minimum inhibitory concentration (MIC) was 0.12 mg/L. The isolate was classified as intermediate (I) to penicillin and susceptible (S) to clindamycin and doxycycline by broth microdilution and was resistant to minocycline. The patient received a total of four weeks of intravenous antibiotics after surgery: two weeks of benzylpenicillin and gentamicin, followed by two weeks of benzylpenicillin. Clindamycin was administered at a dose of 1.2 g IV every six hours because of the severity of disease, susceptibility findings from blood cultures, suspected improved tissue penetration, and the limited published experience in managing *C. diphtheriae* endocarditis. Orthopantomography showed a dental abscess involving the right lower first molar.

**Figure 7:**
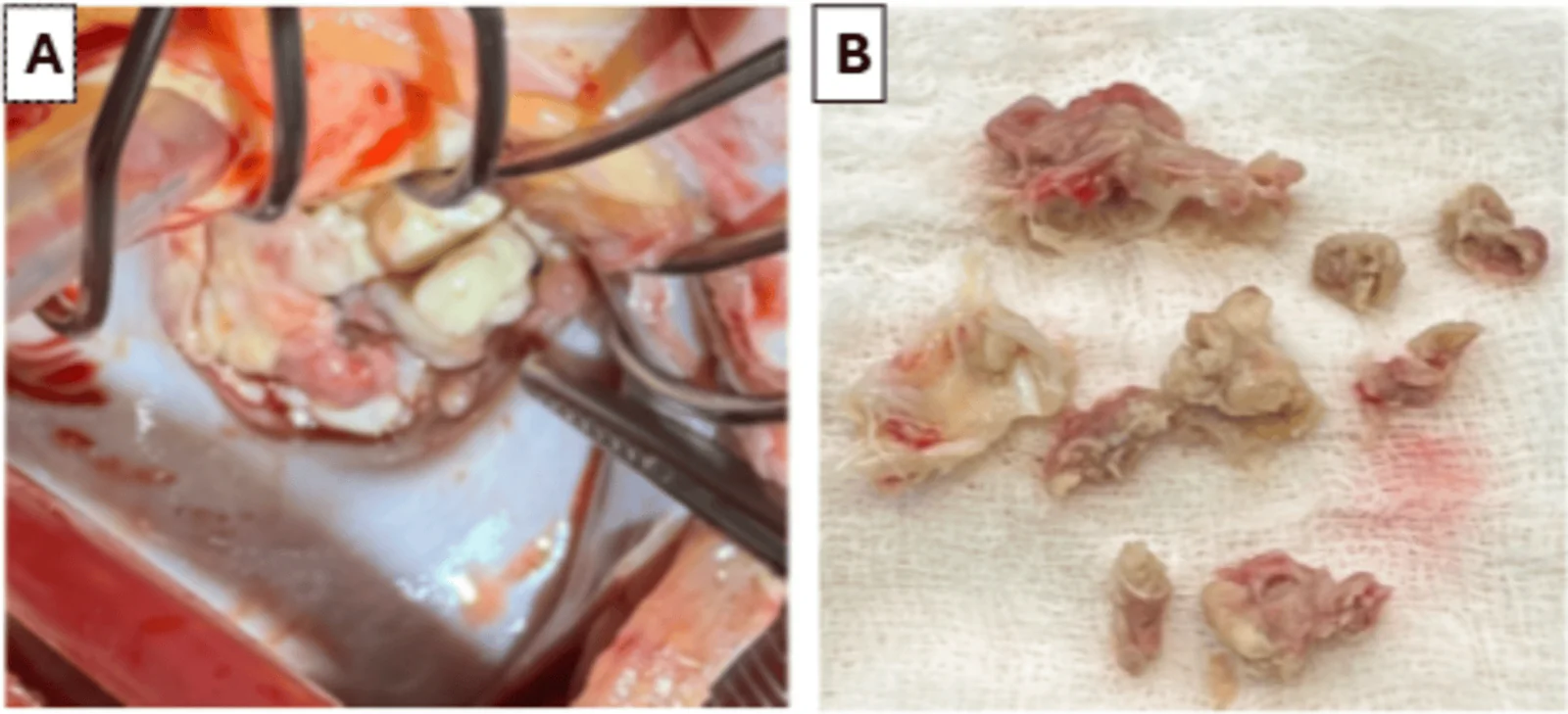
Intraoperative images of the mitral valve (A) Intraoperative image showing the heavily infected mitral valve. (B) Explanted mitral valve showing multiple vegetations attached to the valvular structures.

The patient underwent cardiac surgery within 72 hours of admission. He was found to have heavily infected aortic and mitral valves (Figure 7), with intra-annular extension at the left and noncoronary commissure of the aortic valve and an abscess cavity measuring 1.0×0.5×0.5 cm containing pus. His case was discussed in a multidisciplinary forum with the endocarditis team, and, given concerns regarding adherence to anticoagulation and the risk of ongoing drug use, a decision was made to implant bioprosthetic valves despite his young age, accepting the likelihood of future redo surgery. A 23 mm Edwards Perimount Magna aortic valve and a 29 mm Edwards Perimount mitral valve were implanted, and the abscess cavity was deroofed. The tricuspid valve appeared normal, with thrombus attached to the anterior leaflet that was removed without surgical revision of the native valve. All explanted specimens were sent for microscopy and culture and remained negative after a 56-day incubation period.

The patient completed a four-week course of IV antibiotics as an inpatient and underwent dental extraction postoperatively. He had favorable early postoperative recovery when seen at his four-week post-discharge cardiology follow-up and continued to improve at his three-month post-cardiac surgery outpatient follow-up.

## Discussion

We present a case of native double left-sided valve endocarditis in a young man with an atypical pathogen not commonly considered a cause of infection. This case highlights the well-known challenges in diagnosing endocarditis, as well as the importance of early recognition, collaborative care, and multidisciplinary team (MDT) involvement in achieving good patient outcomes.

The diagnosis of IE is notoriously challenging, largely due to its protean presentation. In 2023, the European Society of Cardiology (ESC) modified the diagnostic criteria to include microbiological pathogens as major criteria and expanded the minor criteria to include additional embolic events and immunological phenomena [7]. The typical causative organisms still include oral streptococci, *Streptococcus gallolyticus*, *S. aureus*, the *Haemophilus* species, *Aggregatibacter* species, *Cardiobacterium hominis*, E*ikenella corrodens*, and *Kingella* species (HACEK) organisms, and *Enterococcus faecalis*. Similar to the Duke criteria [8], predisposing conditions, specifically underlying heart disease or PWID, are considered a minor criterion.

*C. diphtheriae* infects the nasopharynx or subcutaneous tissues and is typically classified into toxigenic and non-toxigenic strains. Toxigenic strains produce a bacterial exotoxin that causes diphtheria, which is typically spread by respiratory droplets or close contact with an infected individual [9]. Immunization against *C. diphtheriae* has led to a dramatic global decline in cases, with associated reductions in morbidity and mortality, and is now recommended by the World Health Organization (WHO) as part of the childhood vaccination schedule [10,11]. However, the vaccine does not prevent infection caused by non-toxigenic strains, which are now emerging [11]. The populations at greatest risk for these strains include people experiencing homelessness, migrants from at-risk countries, and people using inhaled or injectable drugs [12-14]. Non-toxigenic strains can present as acute or chronic wound infections but can also cause rapidly invasive infections such as endocarditis, which has a high mortality rate [4,5,13]. *Corynebacterium* spp. are mentioned in the current ESC guidelines only in the context of cardiovascular implantable electronic device (CIED) infection, which is not applicable to any of the cases reported in the literature to date [7].

A recent case series published by Patel et al. (2025) identified five male patients with IE associated with non-toxigenic *C. diphtheriae* in England who presented between July 2024 and January 2025 [4]. All five patients reported cocaine use by nasal insufflation but no active intravenous drug use and had large vegetations on their initial TTE, as in the present case [4]. Four patients required early surgical intervention, and one died. No patient had documented predisposing conditions according to the modified ESC diagnostic criteria for IE [4]. The UKHSA response led to the retrospective identification of 29 additional cases during the preceding two years in individuals described as having "chaotic" lifestyles who had positive microbiological cultures for C. diphtheriae. These individuals had a range of disease presentations but not endocarditis [15]. These findings highlight the potential underdiagnosis of non-toxigenic *C. diphtheriae* infections in predominantly homeless populations, particularly among people who inhale drugs.

Since 2020, 11 additional cases of non-toxigenic *C. diphtheriae* endocarditis have been described in the literature, occurring in clusters in all but one case [4,5,16-18]. A predisposing cardiac condition was identified in only one patient, and the majority had a history of recreational drug use and unstable housing. The bacterial route of entry is thought to involve damaged nasopharyngeal mucosa resulting from nasal insufflation of cocaine, as suspected in our patient, although this remains a hypothesis given the limited number of reported cases. Seven of the 16 reported cases resulted in death during the index admission.

Of the 16 patients, seven had documented embolic phenomena, and five died. Collectively, these cases demonstrate the virulence of *C. diphtheriae* and its propensity to cause severe endocarditis in patients without classical risk factors.

Our patient met one major criterion (positive imaging for IE) and four of the five minor ESC diagnostic criteria for IE (pyrexia, embolic dissemination, immunological phenomena, and microbiological evidence), but had no "predisposing condition" as defined by the guideline. He received broad-spectrum antibiotics pending blood culture results and was evaluated by the multidisciplinary IE team within 48 hours of admission. Given the severe valvular regurgitation, aortic root endocarditis, and large vegetations with a high risk of further embolization, he underwent emergency cardiac surgery within 72 hours of presentation and was reviewed as an outpatient at four weeks and three months after discharge, continuing to make a good recovery.

The importance of the IE team has been widely demonstrated, resulting not only in earlier diagnosis but also in earlier recognition of complications, appropriate antimicrobial treatment, optimal timing of surgical intervention, and improved outcomes [7]. It is important for noncardiac center hospitals to be aware of IE referral pathways, which often involve tertiary centers that can provide surgical intervention. This is especially true for left-sided disease involving the aortic and mitral valves, which has a high propensity for serious embolic complications, as delays in transfer may limit definitive surgical management.

## Conclusions

Non-toxigenic *C. diphtheriae* continues to pose a significant public health concern, with an elevated risk observed among individuals experiencing unstable housing and substance misuse. These populations are disproportionately affected because of conditions that facilitate disease transmission, including overcrowded or communal living environments, compromised hygiene, and barriers to timely medical care. Although diphtheria remains a notifiable disease under the Public Health (Control of Disease) Act 1984, underreporting within these marginalized groups may contribute to unrecognized outbreaks and further transmission of infection. In the UK, local health protection agencies should be notified of all strains of *C. diphtheriae*, *C. ulcerans*, and *C. pseudotuberculosis*, regardless of toxigenicity.

The severe clinical presentation observed in our patient, consistent with descriptions in the existing literature, highlights the need for heightened clinical vigilance and consideration of endocarditis as a potential complication in socially vulnerable populations, particularly given the high likelihood of requiring early surgical intervention to achieve the best long-term outcome. We also highlight the role of the endocarditis team in improving patient outcomes.
